# Complete Mitochondrial Genome Sequences of Nine Aspergillus flavus Strains

**DOI:** 10.1128/MRA.00971-21

**Published:** 2021-11-11

**Authors:** Miya Hugaboom, Max L. Beck, Katherine H. Carrubba, D. Vignesh Chennupati, Aryan Gupta, Qi Liu, Maya K. Reddy, Fatima Roque, E. Anne Hatmaker

**Affiliations:** a Department of Biological Sciences, Vanderbilt University, Nashville, Tennessee, USA; b Evolutionary Studies Initiative, Vanderbilt University, Nashville, Tennessee, USA; University of California, Riverside

## Abstract

Nuclear genome sequences incompletely characterize the genomic content and thus the genetic diversity of fungal species. Here, we present the complete mitochondrial genome sequences of nine Aspergillus flavus strains, providing useful information for inter- and intraspecific analyses.

## ANNOUNCEMENT

The genus Aspergillus contains over 300 fungal species of varied industrial, agricultural, and medical relevance (1). Aspergillus flavus is a potent producer of aflatoxin B, a carcinogenic mycotoxin and a plant and opportunistic human pathogen (2). Despite the widespread availability of A. flavus genomic sequences, few assembled and annotated mitochondrial genome (mitogenome) sequences are available (3–5). Mitochondrial genes are linked to processes including metabolism, cell differentiation, and drug resistance (6, 7). The assembly and annotation of nine A. flavus strain mitogenome sequences from publicly available sequencing data provides valuable insight into the full genetic profile, evolution, and population genetics of A. flavus.

Genomic DNA was previously isolated and sequenced using an Illumina HiSeq 2500 paired-end 2 × 250-bp platform as described by Drott et al. (8). Paired-end reads from whole-genome sequencing of A. flavus were downloaded from NCBI’s Sequence Read Archive (8), extracted, and split into forward and reverse FASTQ files using SRA Toolkit v2.9.6-1 (9). The reads were trimmed using Trimmomatic v.0.39 (10). Mitogenome sequences were assembled using the specialized genome assembler GetOrganelle v1.7.4.1 (11), with SPAdes v.3.12.0 (12) as the internal assembler. We used the GetOrganelle fungal database (-F fungus_mt) to identify, filter, and assemble target-associated reads with default parameters unless otherwise noted. The complete mitogenome sequence for Aspergillus fumigatus SGAir0713 (GenBank accession number CM016889.1) was used as a reference for the seed database (-s) for each assembly.

A single contig was generated from the GetOrganelle assembly for each strain. Circularization was accomplished via the identification of overlapping nucleotide sequences within the contig FASTA file and the subsequent manual trimming of redundant nucleotides within a text editor. The percentage of mitochondrial reads used for assembly ranged from 1.2 to 2.8% of the total trimmed reads. Read mapping to correct errors was carried out using Bowtie2 v2.3.4.1 (13) and SAMtools v1.6 (14). Bowtie2 was used to align the raw paired-end reads from A. flavus strains against the corresponding circularized mitogenome, and SAMtools was used to identify variants. The read mapping was also visualized and the variants identified using the Integrative Genomics Viewer (IGV) v2.9.4 (15). The mitogenomes had high coverage (830 to 1,300×) when the raw reads were mapped back to the circularized assemblies (Table 1). GeSeq v2.03 (16), a rapid organellar genome annotator, was used to annotate the mitogenomes, with A. flavus (GenBank accession number NC_026920.1), Aspergillus oryzae (NC_008282.1), A. oryzae 3.042 (NC_018100.1), Aspergillus parasiticus (NC_041445.1), and A. fumigatus (NC_017016.1) serving as the references. The gene names were manually revised, and genes with low coverage and sequence similarity (<50%) were discarded. The annotation was finalized following inspection using Geneious Prime v2021.1 (17) to ensure appropriate reading frames and start/stop codons, and the genome sequences were rotated using Geneious to orient the genome start upstream of *cox1*.

**TABLE 1:** Summary of assembly statistics and genomic content for the mitogenomes of nine Aspergillus flavus strains

Strain	No. of raw reads	Total no. of trimmed reads	No. of mitochondrial reads	Length (bp)	Coverage (×)	GC content (%)	No. of tRNA genes	No. of protein-coding genes	*N*_50_ (bp)
FL-B-1-1-1	8,491,957	8,136,443	97,689	29,198	830	26.2	27	16	1
PA-C-1-1-1	4,067,886	3,923,475	110,110	29,323	930	26.1	27	16	1
NC-E-3-2	5,854,147	5,718,609	109,793	29,208	940	26.1	27	16	1
TX-A-2-1-1	8,039,120	7,852,211	104,180	29,204	890	26.2	27	16	1
TX-A-13-1-1	7,750,291	7,599,530	93,233	29,207	890	26.1	27	16	1
TX-A-20-1-1	6,229,075	6,119,690	122,629	29,208	1,050	26.2	27	16	1
TX-A-1-1-1	7,180,878	7,218,213	152,774	29,220	1,300	26.1	27	16	1
TX-B-1-1-1	9,890,328	9,180,887	130,715	29,198	1,120	26.2	27	16	1
TX-B-2-1-1	7,108,842	6,927,904	131,197	29,210	1,120	26.2	27	16	1

All mitogenomes were circular DNA molecules ranging from 29,198 to 29,323 bp with GC contents of 26.1 to 26.2% (Table 1). All mitogenomes contained 16 protein-coding genes, including 14 highly conserved fungal mitochondrial core genes: cytochrome oxidase subunits 1, 2, and 3; NADH dehydrogenase subunits 1, 2, 3, 4, 4L, 5, and 6; ATP synthase subunits 6, 8, and 9; and cytochrome b (3). All mitogenomes also contained ribosomal protein s5 and an intron-encoded LAGLIDADG endonuclease. Two mitochondrial rRNA genes encoding small and large ribosomal subunits and 27 tRNAs were identified in each mitogenome, as expected based on previously assembled Aspergillus mitogenome sequences (3–5).

### Data availability.

The Aspergillus flavus mitogenome sequences from this study are available in GenBank under accession numbers MZ714575.1 (FL-B-1-1-1), MZ714576.1 (PA-C-1-1-1), MZ714577.1 (NC-E-3-2), MZ714578.1 (TX-A-2-1-1), MZ714579.1 (TX-A-13-1-1), MZ714580.1 (TX-A-20-1-1), MZ714581.1 (TX-A-1-1-1), MZ714582.1 (TX-B-1-1-1), and MZ714583.1 (TX-B-2-1-1). The SRA accession numbers for the whole-genome sequencing data used for mitogenome assembly are as follows: SRR12001149 (FL-B-1-1-1), SRR12001150 (PA-C-1-1-1), SRR12001141 (NC-E-3-2), SRR12001147 (TX-A-2-1-1), SRR12001145 (TX-A-13-1-1), SRR12001144 (TX-A-20-1-1), SRR12001148 (TX-A-1-1-1), SRR12001143 (TX-B-1-1-1), and SRR12001142 (TX-B-2-1-1).
